# Waterborne Pathogens: Detection Methods and Challenges

**DOI:** 10.3390/pathogens4020307

**Published:** 2015-05-21

**Authors:** Flor Yazmín Ramírez-Castillo, Abraham Loera-Muro, Mario Jacques, Philippe Garneau, Francisco Javier Avelar-González, Josée Harel, Alma Lilián Guerrero-Barrera

**Affiliations:** 1 Laboratorio de Biología Celular y Tisular, Departamento de Morfología, Centro de Ciencias Básicas, Universidad Autónoma de Aguascalientes, Aguascalientes, Aguascalientes 20131, Mexico; E-Mails: flor.ramirez.castillo@gmail.com (F.Y.R.-C.); aloeramuro@yahoo.com (A.L.-M.); 2 Laboratorio de Ciencias Ambientales, Departamento de Fisiología y Farmacología, Centro de Ciencias Básicas, Universidad Autónoma de Aguascalientes, Aguascalientes, Aguascalientes 20131, Mexico; E-Mail: fjavelar@correo.uaa.mx (F.J.A.-G.); 3 Centre de Recherche en Infectiologie Porcine et Avicole, Faculté de Médecine Vétérinaire, Université de Montréal, St-Hyacinthe, QC J2S 7C6, Canada; E-Mails: mario.jacques@umontreal.ca (M.J.); philippe.garneau@umontreal.ca (P.G.)

**Keywords:** waterborne outbreaks, quantitative risk assessment, viable but non-culturable microorganisms, microbial source-tracking, detection methods

## Abstract

Waterborne pathogens and related diseases are a major public health concern worldwide, not only by the morbidity and mortality that they cause, but by the high cost that represents their prevention and treatment. These diseases are directly related to environmental deterioration and pollution. Despite the continued efforts to maintain water safety, waterborne outbreaks are still reported globally. Proper assessment of pathogens on water and water quality monitoring are key factors for decision-making regarding water distribution systems’ infrastructure, the choice of best water treatment and prevention waterborne outbreaks. Powerful, sensitive and reproducible diagnostic tools are developed to monitor pathogen contamination in water and be able to detect not only cultivable pathogens but also to detect the occurrence of viable but non-culturable microorganisms as well as the presence of pathogens on biofilms. Quantitative microbial risk assessment (QMRA) is a helpful tool to evaluate the scenarios for pathogen contamination that involve surveillance, detection methods, analysis and decision-making. This review aims to present a research outlook on waterborne outbreaks that have occurred in recent years. This review also focuses in the main molecular techniques for detection of waterborne pathogens and the use of QMRA approach to protect public health.

## 1. Introduction

Waterborne disease is a global burden which is estimated to cause more than 2.2 million deaths pear year and higher cases of illness every day, including diarrhea, gastrointestinal diseases and systematic illnesses [1,2]. About 1.4 millions of these deaths are children [1]. It is suggested that waterborne diseases have an economic cost associated of 1 billion dollars annually only in the United States [3]. Worldwide, an economic loss of nearly 12 billion US dollars per year is estimated [4]. Waterborne infections are caused by ingestion, airborne or contact with contaminated water by a variety of infectious agents which includes bacteria, viruses, protozoa and helminths [5]. About 780 million people do not have access to a purified water source, and an estimated 2.5 billion people lack access to improved sanitation worldwide [6]. It is estimated that 3.2% of deaths globally are attributable to unsafe water caused by poor sanitation and hygiene [7]. The United Nations identifies improving water quality as one of the eight Millennium Development Goals (MDGs), and its target is to reduce the number of people without access to safe water by 50% in 2015 [8,9]. The World Health Organization [1,8] has reported that improving water quality can reduce the global disease burden by approximately 4%. Thus, there is an urgent need to undertake all possible efforts to reach this goal.

Even though waterborne outbreaks (WBDOs) have been declining dramatically since the 1900s, the global burden of infectious waterborne disease is still considerable. Moreover, the numbers of outbreaks underestimate the real incidence of waterborne diseases [5]. From 1920 to 2002, at least 1870 outbreaks were associated with drinking water. From 1991 to 2002, 207 WBDOs and 433,947 illnesses, associated with the protozoan agents *Crytosporidium*, *Naegleria fowleri*, *Giardia*, and the bacteria *Salmonella typhimurium*, *Vibrio cholerae*, *Legionella*, *Escherichia coli* O157:H7 and *Campylobacter jejuni*, were reported in the United States of America (USA) [10]. In the period of 1996 to 2006, 21 outbreaks and 507 cases of waterborne disease involving water not intended for dinking were reported. The etiologic agents were *G. intestinales*, *Legionella* spp*.*, *E. coli* O157:H7 and *Pseudomonas aeruginosa* that resulted in acute gastrointestinal illness, acute respiratory illness, hepatitis, dermatitis and several deaths [11]. In 2000, an outbreak in Walkerton, Ontario was linked to the presence of *E. coli* O157:H7 in the Great Lakes area which resulted in 2300 illness cases [12]. From 2001 to 2006, *Legionella* spp., was responsible for 24 drinking water outbreaks, 126 drinking water-related cases and 12 drinking water-related deaths [11]. Between 2007 and 2009, 134 recreational water-associated outbreaks (pools and interactive fountains) were reported by 38 States in USA and Puerto Rico. These caused at least 13,966 cases including 81 outbreaks of acute gastrointestinal illness (12,477 cases), 24 of dermatologic illnesses, and 17 were of acute respiratory illness. Sixty-four percent of the outbreaks were caused by parasites, 21% by bacteria and 4.8% by viruses. The leading etiologic pathogen was *Cryptosporidium* followed by the bacteria *E. coli* O157:H7, *Shigella sonnei*, *Pseudomona*s spp., and *Legionella* spp. [13]. In the Philippines, cholera is an endemic disease; it is estimated that 42,071 cases of cholera have occurred from 2008 to 2013 [14]. In 2010, several outbreaks in Haiti and countries devastated by an earthquake including Dominican Republic and Florida in USA were reported. These outbreaks were related to cholera caused by *V. cholerae* serogroup O1 and O139. The epidemic resulted in around 8534 deaths and 697,256 cholera cases [15,16]. In the same year, around 25 outbreaks associated with drinking water and other non-recreational water were reported in USA. Among the remaining, the leading etiologic agents were the bacteria *Legionella* spp. [13]. Furthermore, in 2011 in Germany, an enteroaggregative Shiga toxin-producing *E. coli* (strain O104:H4) was the causative agent of severe cases of acute diarrhea and bloody diarrhea due to the consumption of uncooked sprouts that were irrigated with contaminated water [17]. In 2012, 18 cases of cholera were reported by European Union (EU) countries. The United Kingdom reported 12 cases, France reported four cases, and Austria and Sweden all reported one case each. In the same year, 9591 cases of *Cryptosporidium* were reported in several countries (United Kingdom, Ireland, Belgium and Germany) largely due to *C. parvum* gp60 subtype IIaA15GR1. A total of 16,368 cases of giardiasis were reported by 23 EU and countries part of the European Economic Area (EEA) such as Bulgaria, Estonia, Sweden and Finland, in 2012. Additionally, 10 waterborne outbreaks were reported in EU during 2012 caused by *E. coli* O157:H7 [18].

Multiple factors produce outbreaks. The infrastructure, chemical coat of pipes and the architecture of the systems could enhance or inhibit the growth of microorganisms even as microbial communities in drinking water systems leading to outbreaks. Breaks or leaks could lead to low pressure events and when repaired pathogens could enter into the systems [3,19]. These failures have been led to outbreaks caused by *Salmonella*, *Campylobacter*, *Shigella*, *E. coli* O157:H7, *Cryptosporidium*, *Giardia* and Norovirus [19,20]. Water treatment deficiencies, such as inadequate or no filtration of surface water and inadequate or interrupted disinfection of groundwater, caused 14% of WBDOs during 2001 to 2002 [10,21]. The weather is another key factor that contributes to outbreaks, since it introduces contaminants into water sources by runoff from either a heavy rainfall or flooding. Moreover, changes in temperature can alter the dynamic of microbes in pipes since planktonic microorganisms may become trapped into biofilms, while pathogens on biofilm may be released in flowing water [3].

Overall, the morbidity and mortality caused by contaminated water are enormous and need to be controlled by improving the security of drinking water [7,8]. In recent years, there have been numerous research advances in methods for detection and characterization of pathogens in water. Detection methods play a major role in monitoring water quality, surveillance, and quantitative microbial risk assessment; thus, have a major influence on implementing the best practices to mitigate and prevent threats that allow achieving the goal of water safety. This review focuses on the new detection methods, principally in molecular methods for detection of waterborne pathogens as well as the use of QMRA to prevent the presence of pathogens in water.

## 2. Emerging Waterborne Pathogens and Related Diseases

Waterborne pathogens have appeared again and again for a number reasons including: contaminated water, increase in sensitive population, changes in drinking water treatment technology, globalization of commerce and travel, and by the development of molecular methods for detection and source tracking [3,8,22]. In developing countries, the lack of financial and technological resources contributes to WBDOs [3]. Currently, it is estimated that there are 1407 species of pathogens infecting humans, which includes bacteria (538 species), viruses (208 types), parasitic protozoa (57 species), and several fungi and helminths species [2,23].

The development of a disease, when and if infection to the host is produced, depends on factors such as minimal infectious dose (MID), pathogenicity, host susceptibility and environmental characteristics. Enteric bacteria have a MID in the range of 10^7^ to 10^8^ cells but it is much lower with some species, such as *Shigella* spp., (10^1^–10^2^), *Campylobacter* spp., (about 500), *E. coli* O157:H7 (10^6^–10^8^), and *V. cholera* (10^3^) [2,5]. Moreover, protozoan only need a few oocysts (10^1^–10^2^) to produce the disease as well as the viruses which a small number of these are enough to develop a disease [2].

The protozoa Microsporidia, as the bacteria *Mycobacterium avium intracellulare*, *Helicobacter pylori*, *Tsukamurella*, *Cystoisospora belli* and viruses such as adenoviruses, parvoviruses, coronaviruses (SARS), and polyomavirus are some examples of the emerging potential waterborne pathogens [22,24]. Furthermore, most of these organisms appear to have certain resistance against chlorine such as the microsporidia, *Enterocytozoon bienusi*, *Encephalitozoon hellem* and *E. intestinales. M. avium* and several viruses have resistance to common disinfectants for drinking water and to inactivation by UV light and heat [22], which represents a higher challenge in the treatment to remove these pathogens from water sources. The major pathogens microorganisms in drinking water systems and their related diseases are listed in Table 1.

## 3. Detection Methods for Waterborne Pathogens

Presently, there is no unified method to encompass the collection and analysis of a water sample for all pathogenic microorganisms of interest [25]. The challenges of the detection methods are the physical differences between the major pathogen groups, low concentration of pathogens in a large volume of water which usually requires enrichment and concentration of the samples prior to detection processing, the presence of inhibitors from the sample (especially if it comes from polluted water), established general protocols for sample collection, culture-independent detection method, as well as detection of the host origin of pathogens [25]. The most important requirements for reliable analysis include: specificity, sensitivity, reproducibility of results, speed, automation and low cost [26].

Even though culture dependent methods are extensively used for pathogens detection in water, these methods are limited by their low sensitivity and the excessive time needed to obtain reliable results. Furthermore, since there is a broad environmental distribution of human pathogens that exist in a viable but non-culturable (VBNC) state such as *E. coli*, *Helicobacter pylori* and *V. cholerae*, false negative results may arise from culture dependent methods [27,28].

**Table 1:** Pathogens in drinking water systems and their related diseases ^a^.

Pathogen	Associated disease ^b^	Health significance ^c^	Persistence in drinking water supplies ^d^
**Bacteria**			
*Campylobacter* spp., *C. jejuni*	Diarrhea, gastroenteritis	High	Moderate
*Yersinia enterocolitica*	Diarrhea, reactive arthritis	High	Long
*Escherichia coli*, particularly enterohemorragic *E. coli* (EHEC), and other such as enteropathogenic (EPEC), enterotoxigenic (ETEC), and enteroinvasive (EIEC)	Acute diarrhea, bloody diarrhea and gastroenteritis	High	Moderate
*Burkholderia pseudomallei*	Meliodosis	High	May multiply
*Legionella pneumophila* and related bacteria	Acute respiratory illness, pneumonia (legionellosis)	High	May multiply
*Non-tuberculous mycobacteria*	Pulmonary disease, skin infection	Low	May multiply
*Pseudomonas aeruginosa*	Infections on lungs, urinary tract, and kidney Can cause inflammation and sepsis	Moderate	May multiply
*Salmonella enterica* serotype Typhi	Typhoid fever, paratyphoid fever and other serious salmonellosis	High	Moderate
*Other salmonellae*	Gastroenteritis, reactive arthritis	High	May multiply
*Shigella spp.*	Bacillary dysentery or shigellosis	High	Short
*Vibrio cholerae*	Gastroenteritis, cholera	High	Short to long
*Helicobacter pylori*	Chronic gastritis, ulcer disease and gastric cancer	Low	Moderate
**Virus**			
Adenovirus	Gastroenteritis	High	Long
Enteroviruses	Gastroenteritis	High	Long
Hepatitis A virus	Hepatitis	High	Long
Hepatitis E virus	Infectious hepatitis; miscarriage and death	High	Long
Rotavirus	Gastroenteritis	High	Long
Sapoviruses	Acute viral gastroenteritis	High	Long
Astroviruses	Diarrhea	High	Long
Norovirus	Gastroenteritis	High	Long
**Protozoa**			
*Acanthamoeba spp.*	Amoebic meningoencephalitis, keratitis, encephalitis	High	May multiply
*Cryptosporidium parvum*	Cryptosporidiosis, diarrhea	High	Long
*Cryptosporidium* *cayetanensis*	Diarrhea	High	Long
*Entamoeba histolytica*	Amoeba dysentery	High	Moderate
*Giardia intestinalis*	Diarrhea	High	Moderate
*Naegleria fowleri*	Infection of the brain called primary amebic meningoencephalitis (PAM)	High	May multiply in warm water
*Toxoplasma gondii*	Toxoplasmosis, miscarriage, birth defects	High	Long
**Helminths**			
*Dracunculus medinensis*	Dracunculiasis (Guinea worm disease, ulcerating skin infection).	High	Moderate
*Schistosoma* spp.	Schistosomiasis, , liver and kidney damage, itchy skin, fever, chills, cough and muscle aches.	High	Short

^a^ Adapted from Table 7.1 in WHO Guidelines for drinking water quality [24]; ^b^ Data obtained from WHO [24], Cabral *et al.* [29], Straub and Chandler [25], and Nygård [30]; ^c^ Health significance relates to the severity of impact, including association with outbreaks; ^d^ Detection period for infective stage in water at 20 °C: short, up to 1 week; moderate, 1 week to 1 month; long, over 1 month.

**Figure 1:**
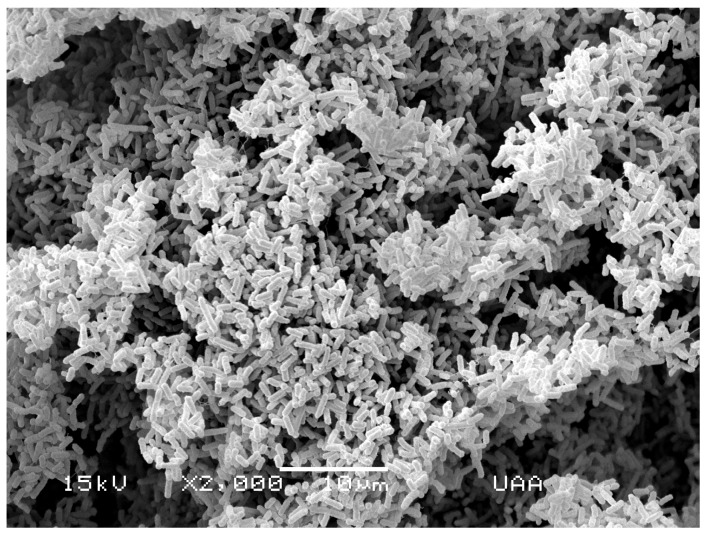
Scanning electron micrograph of *E. coli* isolated from river water.

In both culture and molecular methods, index pathogens for monitoring water quality have been selected in order to indicate the presence of a large amount of pathogens in water. Among these, *E. coli* (Figure 1) has been extensively used due to the fact that detection methods for these pathogens are relatively easy and inexpensive; nonetheless, they may have the disadvantage of not providing information on their host origin and, sometimes, they do not correlate with other pathogens present in the water, such as the viruses and protozoa. Thus, water characterized as pathogen-free by monitoring *E. coli,* for example, may be contaminated with viruses or protozoa [31].

Molecular methods can be very specific for particular species and provide further phylogenetic information about pathogens [32]. These methods allow the use of alternatives indicators which easily relate with the host source. This permits the discrimination between human and animal pathogens and tracking the source of pollution [29,31]. It has been suggested that host–origin libraries, based on a phenotypic methods, may be useful for tracking the pathogen source but the development of such libraries may incur a significant cost [8]. Therefore, molecular methods seem to better suited for health risk assessments.

Nowadays, there are a number of different molecular methods to detect diverse pathogens. They are used to evaluate the microbiological quality of water, the efficiency of pathogens removal in drinking and wastewater treatment plants, and as microbial source-tracking (MST) tools [33]. Several examples of detection methods and their limits of detection are listed in Table 2. Molecular techniques, such as nucleic acid amplification procedures, offer sensitive and analytical tools for detecting a variety of pathogens, including new emerging strains, present the possibility of automation, and real-time analysis to provide information for microbial risk assessment purposes [33].

**Table 2:** Application of detection methods for pathogens and their detection limits.

Detection Method	Water Pathogens	Detection Limit (LOD)	Matrix Sample	References
PCR	*E. coli*	1 cfu /100 mL	Contaminated tap water.	[34]
	Enterotoxigenic *E. coli* (ETEC)	4 cfu/mL	Water samples spiked by ETEC and nonpathogenic *E. coli*.	[35]
	*Cryptosporidium* and *Giardia*	1 to 10 oocyst and 5–50 cysts	Environmental water samples.	[36]
Multiplex PCR	EHEC, *Shigella sp.*, *Vibrio parahaemolyticus*, *P. aeruginosa* and *Salmonella sp.*	10^1^ cfu, 10^2^ cfu, 10^2^ cfu, 10^2^ cfu and 10^1^ cfu	Polluted water and natural water.	[37]
Quantitative PCR (qPCR)	Adenovirus (adenovirus fiber gene in AdV40 and AdV41)	5–8 copies of AdV40/41	Wastewater, drinking water, recreational waters, and rivers.	[38]
	*L. monocytogenes*, *V. cholerae*, *V. parahaemolyticus*, *Pseudogulbenkiana* sp., *S. typhimurium*, *S. flexneri*, *C. perfringens* and pathogenic *E. coli* (Microfluidic qPCR)	From 10^2^ to 10^4^ cells per ca. 200 mg fecal samples of pathogens 100 cells/L	Spiked environmental water samples (pond) and natural fresh water lake.	[39]
	Adenovirus, Aichi virus, astrovirus, enterovirus, human norovirus, rotavirus, sapovirus, and hepatitis A and E viruses (Microfluidic qPCR)	2 copies/μL of cDNA/DNA	River water contaminated with effluents from a wastewater treatment plant.	[40]
Real-time PCR	*V. cholerae*	1 cfu/100 mL	Ballast water.	[41]
	*C. hominis, C. parvum, C. meleagridis, C. wrairi*	1 oocyst	Sewage and river water.	[42,43]
	Astrovirus	5–7 GC logs /100 mL	Sewage.	[44]
	*L. monocytogenes* on biofilms	6 × 10^2^ cfu/cm^2^	Artificial biofilms.	[45]
Microarrays	*Bacillus anthracis, Brucella abortus, C. botulinum*, *Coxiella burnetii*, *Francisella tularensi, Rickettsia prowazekii, C. perfringens, S. aureus*, *V. cholerae*, *V. alginolyticus*, *Yersinia pestis* Western equine encephalitis, Eastern equine encephalitis, Ebola, Venezuelan equine encephalitis virus, *Alexandrium cantenella*, *Fusarium sporotrichioides*	10 fg of *B. anthracis* DNA, 500 fg of *F. tularensis* DNA and *Y. pestis DNA*	Environmental water and ocean water spiked with pathogen.	[46]
	*Y. enterocolitica*, *E. coli, S.enterica* Typhimurium,	1 × 10^7^ *S.* *typhimurium* cells (10^4^ *S. enterica* genomes)	Wastewater.	[47]
	*Cryptosporidium, Acanthamoeba* spp., *Blastocystis hominis*, Entamoeba spp., *Giardia intestinalis*, *Naegleria* spp.	1 × 10^3^ target genes, or 50 *Cryptosporidium parvum* oocysts, per assay	Municipal wastewater treatment plants.	[48]
Microarray	*E. coli*, *A. hydrophila* (DNA microarray and real-time qPCR techniques)	10^3^ *A. hydrophila* cells per sample, 5 ng of *E. coli* and 10 ng of *A. hydrophila*	Municipal wastewater treatment plant in each stage of the disinfection process.	[49]
	*C. parvum*, *C. hominis*, *E. faecium*, *B. anthracis*, *F. tularensis*	100 ng of DNA, Microarray assay: 20 genomic copies without a PCR pre-amplification step	Tap water spiked with multiple organisms.	[50]
Pyrosequencing	*Y. pestis*	0.9 cfu/mL	Milk.	[51]
	*B. anthracis* (Immunomagnetic separation and pyrosequencing)	6 cfu/mL	Bottled water, milk and juice.	[52]
	Comamonadaceae, Proteobacteria, Bacteroidetes, Planctomycetes, and *Elusimicrobia*	1.3 ×10^5^ cells/mL	Drinking water of the non-chlorinated distribution system.	[53]
Biosensors	*C. parvum*	10^5^ oocysts/mL	Oocysts diluted in PBS.	[54]
	*E. coli 0157:H7* (NASBA +LFA biosensor)	40 cells/mL	Drinking water.	[55]
	*V. cholerae* (Amperometric)	8 cfu/mL	Ground and sea water.	[56]
	*Microcystis spp.* (Optical fibre)	30 ng/L	Lake water.	[57,58]
Fluorescence *in situ* hybridization (FISH)	*F. psychrophilum*	7.3 × 10^5^ cells/mL	Culture suspension.	[59]
	*E. coli* (catalyzed reporter deposition (CARD)-FISH)	8.9 ±1.5 16S rRNA molecules per cell	Mixed pure cultures and sludge.	[60]
	*E. coli*	1,400 ± 170 16S rRNA copies per *E. coli*	Activated sludge.	[60]
Immunology-based methods	*E. coli* O157:H7 and *S. enterica* typhimurium.	1.8 × 10^3^ cfu/mL of *E. coli* and 9.2 × 10^3^ cfu/mL of *Salmonella*	Contaminated food.	[61,62]

* GC logs are mean values of genome copy logs.

### 3.1. Polymerase Chain Reaction (PCR)

Polymerase chain reaction (PCR) is one of the most commonly used molecular-based methods for detection of waterborne pathogens [28]. PCR operates by amplifying a specific target DNA sequence in a cyclic three-step process—denaturalization, annealing and extension—in order to achieve exponential amplification of the target sequence [63]. Several variations of PCR such as multiplex PCR (mPCR) allow simultaneous detection of several target organisms by coding specific genes of diverse pathogens in the sample in a single reaction. PCR method has the advantage of quick analysis. However, it necessitates accurate primers and optimal reaction mixture to avoid the risk of false positive and negative results [28,33].

Limitations of DNA based methods such as PCR include the inability to discriminate between viable from non-viable cells that both contain DNA, the low concentration of several pathogens in water such as *Cryptosporidium*, *Giardia* and viruses, and the lack of data to indicate the real infectious risk to a population. Challenges of molecular techniques include: the need for water concentration methods (*i.e.*, for virus, ultrafiltration and direct flocculation protocols are used as concentration methods), the presence of inhibitors in water samples including humid acids and metals, to which PCR is sensitive, and the reproducible purification techniques by DNA or RNA from heterogeneous samples. In addition, result validation is required, since as indicated by Hartman and co-workers [64]; also, high sensitivity in molecular techniques introduces a high risk of false positive results.

An example of mPCR includes the technique developed by Omar and Barnard [65] to detect the pathogenic and commensal *E. coli* from clinical and environmental water sources. To distinguish pathogenic *E. coli* from commensal, the presence of 11 genes was evaluated in environmental waters. In addition, as control to evaluate the sensitivity of the technique and the false negative due to PCR, inhibitors were controlled using *mdh* gene (malate dehydrogenase) and *gapdh* gene (glyceraldehyde-3-phosphate dehydrogenase).

Another variation of the PCR is quantitative real-time PCR (qPCR) which enables quantification of DNA targets by monitoring amplified products during cycling as indicated by increasing fluorescence [66]. This method provides high sensitivity and specificity, faster rate of detection, minimizes the risk of cross-contamination, and there is no need for a post-PCR analysis [67]. The dual-labeled fluorescent probes such as TaqMan probe and the fluorescent dye SYBR green are the most used techniques for detecting pathogens [68]. qPCR approach has been used to quantify human pathogens, such as *E. coli* O157:H7 [69], and *Campylobacter* spp., [70]. These qPCR systems can specifically detect and quantify pathogens at concentrations as low as one target molecule per reaction. However, most of these methods can only detect and quantify one pathogen in a single reaction [71]. Ishii and co-workers [39,71] designed a microfluidic qPCR by using TaqMan probes (hydrolysis probe-based qPCR) labeled with different fluorophores that can detect *L. monocytogenes*, *V. cholerae*, *V. parahaemolyticus*, *Pseudogulbenkiana* sp., *S. typhimurium*, *S. flexneri*, *C. perfringens* and pathogenic *E. coli* at levels of detection of 100 cells/L. Other advances in PCR include the microfluidics and nanobiotechnology field, allowing the construction of high-density and low-volume qPCR platforms, such as the OpenArray system that accommodates 3072 reactions per array [72].

qPCR protocols have also been developed for the detection and identification of *Cryptosporidium* spp., in river water with a lower quantitation limit of 2.5 oocysts/sample [42]. For detection of *G. lamblia* and *G. ardeae* in wastewater samples, qPCR reached a sensitivity of 0.45 cysts per reaction [73]. Moreover, because of the importance of biofilm coating pipes in drinking water systems, the detection of pathogens in microbial communities is important. *L. monocytogenes* has been investigated in biofilms by using qPCR techniques with a number of *L. monocytogenes* detected growing in biofilm of 6 × 10^2^ cfu/cm^2^ [45]. For RNA virus detection, quantitative reverse-transcriptase (qRT)-PCR was developed in order to provide quantitative estimation of the concentration of pathogens in water [74]. This technique has the advantages of detecting viable cells due to detect messenger RNA (mRNA), which is present only in viable organisms. However, damaged genomes may fail to be detected with this technique [32,75].

### 3.2. Oligonucleotide DNA Microarrays

Oligonucleotide microarrays are a powerful genomic technology that is widely utilized to monitor gene expression under different cell growth conditions, detecting specific mutations in DNA sequences and characterizing microorganisms in environmental samples [76]. DNA microarrays are arrays containing high density immobilized nucleic acids (genomic DNA, cDNA or oligonucleotides) in an ordered two-dimensional matrix that enables the simultaneous detection of hundreds of genes in a single assay via nucleic acid hybridization [77]. Microarrays are made up of glass slides or chips coated with specific oligonucleotide probes which are chemically synthesized short sequences (25 to 80 bp) [28,78]. Microarray technology allows the rapid detection of multiple genes of multiple organisms simultaneously in the sample due its capacity for screening large numbers of sequences [32], has high throughput capacity and is able to be automated. Therefore, large-scale and data-intensive experiments are performed in microarrays [4,28]. Microarrays have also the ability to detect antimicrobial resistance to different antibiotics, and the probes could be designed for detecting the host origin of contaminants, which represents another advantage for characterization of contamination in water. However, advanced molecular technologies such as microarrays could experience difficulties distinguishing between viable and non-viable cells, have a relatively high cost, and may have non-specific hybridization resulting in a lower specificity and low sensitivity [32].

DNA microarray coupled with PCR amplification of target genes has been developed for higher sensitivity. Through these steps, signal sensitivity increased around 10^6^-fold [79]. Nonetheless, the PCR-microarray technique might lack sensitivity when the amount of sample is limited, since the technique requires splitting the samples into several PCR reactions to amplify [50]. DNA microarray in a two-step PCR-DNA microarray assay that first amplifies the 16S or 18S rRNA gene, respectively, followed by hybridization of these products onto a low-density DNA microarray was developed to detect most of the common waterborne protozoan pathogens including *C. parvum*, *G. intestinalis*, *Acanthamoeba* spp., *Entamoeba histolytica*, and *Naegleria* spp. [48,49]. In 2002, Wilson and co-workers [46] were able to identify 18 pathogenic bacteria, eukaryotes and viruses by using species-specific primer sets to amplify multiple diagnostic regions unique to each individual pathogen in the microarray. Inoue and co-workers [80] studied the occurrence of 941 pathogenic bacterial species in groundwater and were able to distinguish between human and animal sources.

Microarrays for detection of human enteric viruses in community wastewaters using cell culture coupled with multiple targets including DNA and RNA viruses have also been developed [81]. The ViroChip, a pan-virus DNA microarray containing thousands of 70-mer oligonucleotide probes to target all viral families to infect humans, has been described by Wang and co-workers [82]. Leski *et al.* [83] developed a high-density re-sequencing microarray that has the capability of detecting 84 different types of pathogens ranging from bacteria, protozoa, and viruses, including *B. anthracis*, *Francisella* *tularensis* and Ebola virus with limits of detection of 10^4^ to 10^6^ copies per test for nearly all the pathogens with high specificity.

At present, standard and custom-made DNA microarrays are commercially available from companies such as Affymetrix, Corning and Agilent Technologies [84,85]. One example of these is the phylogenetic microarray “PhyloChip” by Affymetrix which consists of 500,000 oligonucleotide probes capable of identifying 8743 strains of bacteria and Archaea [86,87]. This technology is very powerful because most known bacteria can be detected in samples without culturing, and the sensitivity allows also the detection of lower-abundance organisms (detection limit of ~0.01% of microbial communities) [88].

### 3.3. Pyrosequencing

Pyrosequencing is a DNA sequencing technology for short-read DNA sequencing that uses enzyme-couple reaction and bioluminescence to monitor the pyrophosphate release accompanying nucleotide incorporation, in real time [89]. Pyrosequencing technology provides a large number of sequences read in a single run [90]. In the pyrosequencing reaction, the enzymes DNA polymerase, ATP sulfurylase, luciferase and apyrase are needed. The nucleotides are added to form the complementary strand of the single-stranded template, to which a sequencing primer is annealed. During the nucleotide incorporation, the pyrophosphate (PPi) is released. ATP sulfurylase converts the PPi into ATP and the ATP is then converted to visible light by luciferase and the produced light signal is detected [87,90]. The methodology includes a primary step of concentrating the water pathogens followed by a secondary concentration depending of the type of the pathogen. The third step involves DNA extraction (for bacteria and protozoa) and nuclease treatment (viral DNA/RNA extraction). Then, the DNA is amplified, the pyrosequencing is taking place and finally the analysis and bioinformatics can be performed [87,90].

In recent years, a new whole genome amplification and sequencing approach called “Single Virus Genomics”, which enables the isolation and complete genome sequencing by 454 pyrosequencing of the single virus particle, has been described [91]. Roche 454 pyrosequencing and other commercially available high-throughput sequencing platforms such as Solexa/Illumina Genome Analyzer, Applied Biosystem SOLiD Sequencing as well as the most recent, the Ion Torrent system, have revolutionized the study of microbial diversity [87].

Pyrosequencing could identify novel pathogens associated with water and address multiple etiologies. However, the DNA amount present in wastewater samples could limit the sensitivity of this tool as it requires DNA templates at picomole level but a much lower amount of DNA may be available in water samples. This technology is also limited by the cost, the complexity of analysis, the need for increasing availability of massive computing power and the efficiency of data generation [92].

Pyrosequencing has been used to the analysis of bacterial biofilm communities in water meters of a drinking water distribution system [93], mixed urban watershed [94], characterization of nontuberculous *Mycobacterium* communities in unchlorinated drinking water [95], and the detection of bacteriophages in reclaimed and potable and lake waters [96].

### 3.4. Fluorescence *in Situ* Hybridization *(FISH)*

**Figure 2:**
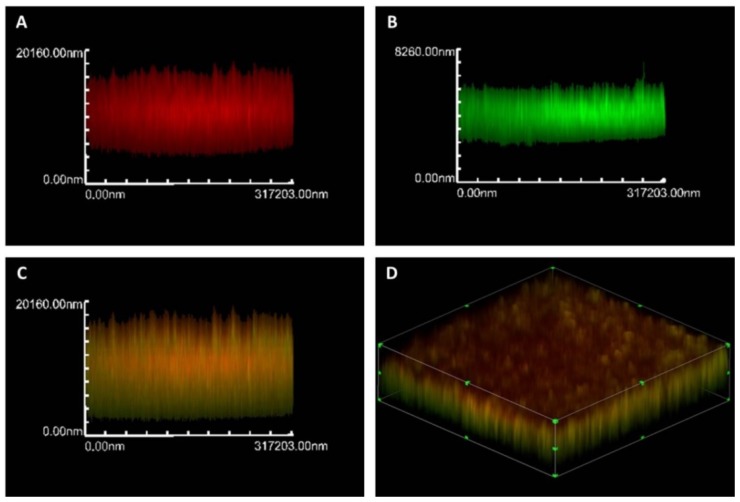
Confocal scanning laser microscopic images. (**A**) *Actinobacillus pleuropneumoniae* isolated from water sources in the di-species biofilm of *A. pleuropneumoniae* 719 and *S. suis* 276 by FISH with an ApxIVAN-AlexaFluor 633 probe (red); (**B**) Bacterial cell in the biofilms were stained with FilmTracer ™ FM ^®^ 1-43 (Molecular Probes) which are represented in green; (**C**) Yellow represents the co-localization of both the ApxIVAN probe and the stain FM 1-43; (**D**) 3D images of biofilm dual-species biofilms grown in BHI with NAD.

FISH or fluorescence *in situ* hybridization is based on hybridization of the sample with rRNA oligonucleotide probes labeled covalently at one end with fluorescent dye. This technique allows an enumeration of particular microbial cells by the use of confocal microscopy, fluorescence microscopy or flow cytometry in order to obtain qualitative and quantitative results [97]. FISH is commonly used for the detection and identification of different microorganism in mixed populations such as in biofilms (Figure 2) and has been used to produce a quantitative description of the microbial community structure in activated sludge and wastewater [79,98], to study mechanisms of survival, infection at cellular level and detection of emerging pathogens from water, sewage and sludge [79]. One example is the two-color FISH assay, based on species-specific probes for *C. parvum* (Cpar 677 probe) and *C. hominis* (Chom253 probe), and has been shown to distinguish between the two species of concern to public health [33,99].

Several kits of FISH are now available in the market, one example of these are the commercial kit *Label* IT ^®^ Fluorescence *In Situ* Hybridization Kit Cy^®^3, fluorescein and TM-Rhodamine, which optimized the preparation and hybridization of fluorescently labeled DNA probes [100].

Viable But Non-culturable (VBNC) cells could detected by FISH couple with direct viable count (DVC) assay [101,102,103]. In the assay, the cells are cultured in reach media with antibiotics which prevent cellular division, increases of intracellular rRNA and allow the elongations of viable cells. FISH is made with specific probes that target 16rRNA labeled with fluorescent dye. The sample is stained by 4,-diamino-2-pheynyl indole (DAPI) that is bound to double-stranded DNA to count the total amount of cells in the samples, and the results are compared to culture methods to measure the proportion of VBNC cells [103]. Members of the family *Enterobacteriaceae* and *E. coli* in drinking water systems, freshwater and river water have been detected by this tool [103].

This assay is able to detect 3000 viable *E. coli*/100 mL in more than 10^8^ non-*E. coli*/100 mL [103]. However, the technology is limited by a low sensitivity, and the necessity of pre-enrichment and concentration steps [33] that also may increase inhibitor concentrations and lead to false negative results [32].

VBNC cells are living cells, metabolically active, that may still retain or regain their virulence potential upon the resuscitation (return to culturable state) under favourable environmental conditions or when the stress is removed [104,105,106,107]. Due to the fact that VBNC cells cannot be identified by culturable methods, the number of pathogens in this state could be underestimated and, if all bacteria in the sample are in VBNC state, the sample may be regarded as pathogen-free due to non-detection [105]. The conversion to the VBNC state is supposed to be a response to adverse environmental conditions [105,106,107]. Resuscitation of these species could be triggered by a variety of stimuli, such as rich medium, an increase in temperature, and the presence of host cells. Factors as the strain used, the age of VBNC cells, and the conditions that induced the VBNC state also affect the resuscitation [105,108,109].

Besides FISH, other tools to detect VBNC bacteria includes: immunological techniques; RT-PCR, which target the mRNA that indicates the presence of viable cells that carry out transcription; DNase I protection assay, since only the viable cells have intact membranes to protect genomic DNA from digestion by exogenous nucleases; enzymatic activity, as viable cells carry out metabolic reactions and respiration; bacteriophage-based assay, using a combination of fluorescence intensity and nutrient uptake analysis; as well as the commercial kit LIVE/DEAD ^®^ *Bac*Light^TM^ assay to distinguish viable cells from dead cells based on the membrane integrity [102,103,104,105,106,107,108,109].

### 3.5. Immunology-Based Methods

Immunological detection with antibodies is a technology that has been employed for the detection of multiple pathogens [110,111]. These methods are based on antibody-antigen interaction, whereby a particular antibody will bind to specific antigen which comprise the use of polyclonal and monoclonal antibodies [28,63]. Immunological detection includes, for example, serum neutralization tests (SNT), immunofluorescence, and enzyme-linked immunosorbent assays (ELISA). SNT test has been used for the serotyping of viruses and involves mixing a sample, extracted from a plaque assay, with antiserum and then assessing the decrease of infectivity by plaque assay [112]. Assays based on immunofluorescence and immunomagnetic separation (IMS) are used to detect protozoan parasites. The US Environment Protection Agent (EPA) has established method 1623.1 for the combined detection of *Cryptosporidium* spp. and *Giardia* spp., or method 1622 for *Cryptosporidium* spp., both of which use IMS followed by immunostaining and fluorescent microscopy [113,114]. Nevertheless, limitation such as low sensitivity, false negative results, cross-reactivity with closely related antigens and the need for a pre-enrichment in order to reduce the cell surface antigens, are present in these techniques [33].

### 3.6. Biosensor Based Methods

Biosensor based methods have flourished in recent years. Biosensor is an analytical device that consists of a bioreceptor that recognizes the target analyte (e.g., enzymes, antibodies, nucleic acids, cell receptors, aptameters, recombinant antibodies, imprinted polymers and synthetic catalyst) and a transducer that converts the biological interactions into a measurable electrical signal (optical, electrochemical, mass-based, thermometric, or micromechanical) [26,33,112], thereby providing selective quantitative or semiquantitative analytical information [114].

Optical biosensors rely on a change in the optical properties of the surface caused by the binding of the analytes used for detection [114]. Electrochemical biosensors are based on measuring changes in conductance, resistance or capacitance of the active surface. In these devices, one of the electrodes is immobilized with a recognition molecule. When analytes bind, a change in electrical properties occurs providing the sensor signal [115,116]. Mass-sensitive biosensors include quartz crystal microbalance biosensor, which uses a quartz crystal inserted in two electrodes. As quartz is piezoelectric (used for wave propagation), the crystal can be excited by applying a voltage across the electrodes and will exhibit a resonance frequency [115,117].

Biosensor methods have the advantages of automation and miniaturization of biological analytical techniques, have short analysis times, portability, real-time measurements and do not require sample pre-enrichment [117]. Nevertheless, problems such as great sensitivity to pH, change of mass, temperature, *etc.*, represent a great challenge to the use of these methods [115].

In 2005, Taylor and co-workers [118] used the surface plasmon resonance (SPR) detection with pretreatment of the bacteria (heat or ethanol treated or detergent-lysed cells) and the antibodies goat anti-*E.coli* O157:H7 polyclonal antibody (PAb) and mouse anti-*E. coli* O157:H7 monoclonal antibody (MAb) for signal amplification to obtain a limit of detection (LOD) of 10^4^ cfu/mL. Antibody-based indirect sensor (optical biosensor) for *Escherichia coli* O157:H7 with fluorescent labeling was reported by Ho *et al.*, [119] in 2004 to reach a LOD of 360 cells/mL.

Detection and monitoring of pathogens in water continues to be a field with constant improvement and development of new tools that will permit relative low cost assays and rapid identification of multiple pathogens with limits of detection that meet regulatory goals. Nevertheless, efforts to standardize techniques in the field have to include sample collection, sample concentration, sample purification, sample processing, analysis, and data collection are still remaining [3]. Sample collection has to be standardizing mainly if the target is on biofilms. Validation of new emerging techniques has also to be completed.

Besides microarrays and biosensors, the integration of molecular technologies is promising. “Lab-on-chip” (LOC) represents an integration of sensors and microfluidic systems in a miniaturized tool that could archive real-time monitoring of samples [120]. By this way, LOC integrates several laboratory functions on a single chip and reduced sample and reagent consumption is automatized and has fast detection times and low limits of detection. However, there are still challenges related to developing practical LOC components, assay procedures and validation of the on-chip detection approaches, and of course, the different scenarios that represent a water sample [121].

## 4. Quantitative Microbial Risk Assessment of Waterborne Disease

Since outbreaks continue to occur despite water quality monitoring, a new approach has been applied to solve the need for a conceptualization of risk and to provide guidance and legislations [122]. Quantitative microbial risk assessment (QMRA) is a systematic quantitative assessment process to estimate the health risks or illness rates of human exposure to particular pathogens. The approach combines dose response information for the infectious agent with information on the distribution of exposures routes and is able to demonstrate if the health targets are met in a drinking water distribution system [123], which are suggested to be a health risk less than one infection per 10,000 individuals per year, or, a disease burden of 10^−6^ disability adjusted life year (DALY) per person per year [3]. This tool helps to predict the burden of waterborne diseases, set tolerable limits of waterborne disease, identify the means to reduce the risk to the consumers and determine measures to protect water safety [2].

**Figure 3:**
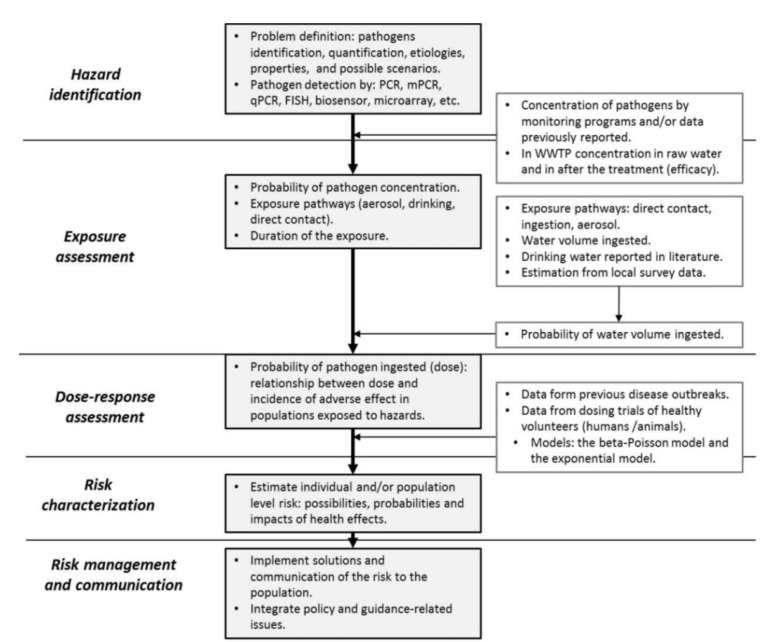
General approach for quantitative microbial risk assessment (QMRA). WWTP, wastewater treatment plant.

Risk assessments involve five steps (Figure 3) [2,124,125,126,127]. (i) *Hazard identification*: consists in the identification and quality evaluation of the microbial hazard. This step takes into account the potential outcomes (health effects, disease outbreaks), pathogen properties (virulence, adaptation, resistance, and mutation) and hazard pathways; (ii) *Exposure assessment*: is the estimation of the duration of human exposures to pathogens by specific routes, the determination of the size and nature of the population exposed and the barrier reduction and recontamination on water; (iii) *Dose-response assessment* is the characterization of the relationship between dose and incidence of adverse effect in populations exposed to microbial pathogens. It comprises data of illness, secondary transmission and immunity in population. Typically, the dose-response is derived from studies of exposure of human volunteers to different doses of the pathogen or is based on previous outbreaks [127,128,129]. Factors influencing the dose-response are the exposure route, exposure medium pathogen strain host endpoint and the data source. Two commonly models are the beta-Poisson model and the exponential model, described elsewhere [2,125,130]; (iv) *Risk characterization* is the integration of information from hazard identification, dose-response assessment, and exposure assessment to estimate the magnitude of health effects; (v) *Risk management and communication* is a decision-making process based in all previous steps in risk assessment [2,125,126,127,128].

In QMRA, the different variants contribute to measuring the health risk but also introduce variability corresponding to dose-response sensitivity, detection methods, temporal and spatial heterogeneities in pathogen densities and uncertainty (*i.e.*, unrepresentative population, modeling pathogen densities and knowledge of dose–response relationships) [124,130,131,132]. The advantage of probabilistic modeling is to distribute the uncertainties through the model [133]. The QMRA approach presents potential advantages and limitations of risk management options that could be evaluate by numerical stimulations to investigate their possible efficacy, and that risk below epidemiologically detectable levels may be estimated under specific circumstances [134]. However, the greatest limitation on QMRA is the lack of available data to carry out this assessment [134].

QMRA also depends on the method used for monitoring pathogens in water. Actually, QMRA approach is based exclusively on data obtained by cultivation methods, which are not allowing the complete characterization of risk, since as mentioned above, many waterborne microorganisms are not culturable or are inefficiently cultured, thus leading to underestimation of pathogens in water. Therefore, selecting the optimal tool for pathogen monitoring in source water that gives the necessary information on occurrence, prevalence, virulence, and even viability of the pathogens influences the results of the QMRA approach.

Polymerase chain reaction methods provide rapid detection of microorganisms but are complicated by the presence of inhibitory compounds in the sample and the detection of viable but non-culturable as well as non-viable pathogens. Integrating the culture and PCR methods may help to overcome the weakness of each individual method [133,135,136].

As mentioned before, current risk characterization approaches rely on detection of microbial indicators or “index pathogens”, which does not accurately predict the occurrence of all pathogens. Novel markers that better predict the presence of all pathogens of interest would improve risk assessment and management actions [31,123]. Alternative indicators proposed are *Bacteroidales* and *Lachnospiraceae*, which are rich in host-specific bacterial organisms [31,137], faecal bacteriophages, rotavirus, *Ascaris* [138], Firmicutes [31], *Bifidobacteria* spp. [139], and *Methanobrevibacter smithii* [140]. Commonly 16S rRNA gene has been used as a marker to track host-specific organisms [31], but other authors such as Villemur *et al.* [141] and Caldwell *et al.* [142] suggest the use of DNA (mtDNA) to target the identification of human and animal origins.

QMRA has been used to estimate the health risk associated with bathers after surfing and swimming in dry weather and post-storm conditions near beaches [143,144,145], water distribution networks [146], recreational waters impacted by agricultural contamination [122,147], and assessment of the efficiency of the applied treatment processes in drinking water [143]. Such investigations reinforce the need for preventive measures, such as those designed to prevent and take measures to reduce water contamination. A computational tool for quantitative microbial risk assessment, called QMRAspot, was developed for rapid and automatic undertaking of QMRA for drinking water. QMRAspot uses four index pathogens: *Campylobacter*, enterovirus, *Cryptosporidium*, and *Giardia* [143]. Also, a software infrastructure name FRAMEs (Framework for Risk Analysis in Multimedia Environmental Systems) have been created to assess the water health risk which allows describing the problem statement, integrating models and data-bases, and provides the infrastructure for performing sensitivity, variability and uncertain analyses [131].

It is clear that QMRA and management of water quality are important tools for governmental regulation and scientific analysis. Nevertheless, it is also clear that these tools still are not adopted widely [123]. Efforts to encourage the use of the QMRA approach have to be made to support implementation of water safety plans, improve understanding of vulnerabilities of drinking water distribution systems, assess risks associated with extreme events and establish the best management option for the given risk in order to ensure water safety [3].

## 5. Conclusions

Waterborne pathogens are a global concern for worldwide public health. Since pathogens in water are still a major cause of severe illness and mortality, the control, monitoring and application of regulations for water quality are in urgent need and must incorporate more effective microbiological monitoring, pathogen detection and health risk assessment in order to reach the goal of pathogen-free water.

Although culture methods for detection of pathogens in water are used routinely, they may underestimate the level of microbial pathogens. Molecular techniques improve the characterization of these pathogens; however, several disadvantages such as the lack of standardization of protocols and sample processing are still a challenge. Improving available technologies so that they are able to identify causative agents more accurately and in a shorter amount of time, to detect viable microorganisms and characterize them according to microbial communities and that enable the creation of accessible data could enhance the knowledge of waterborne pathogens and the possibilities to predict pathogen contamination and protect public health.

Pathogen indicators need to be continually improved since a large number of new emerging pathogens are causing water-related diseases and waterborne outbreaks. The implementation of QMRA needs to be adapted to estimate the level of risk from different pathogens for better understanding of the dynamics of microbial populations in drinking water systems, and to identify the most effective strategies to be implemented to reduce the health risk and to improve water quality.
